# Flow Direction-Dependent Elastic Instability in a Symmetry-Breaking Microchannel

**DOI:** 10.3390/mi12101139

**Published:** 2021-09-23

**Authors:** Wu Zhang, Zihuang Wang, Meng Zhang, Jiahan Lin, Weiqian Chen, Yuhong Hu, Shuzhou Li

**Affiliations:** 1 College of Physical and Material Engineering, Guangzhou University, Guangzhou 510006, China; 1919400068@e.gzhu.edu.cn (Z.W.); 1619200027@e.gzhu.edu.cn (J.L.); wqchen@e.gzhu.edu.cn (W.C.); 1919400035@e.gzhu.edu.cn (Y.H.); 2 Precision Medicine Institute, the First Affiliated Hospital of Sun Yat-Sen University, Sun Yat-Sen University, Guangzhou 510080, China; meng.zhang_china@outlook.com; 3 School of Materials Science and Engineering, Nanyang Technological University, Singapore 639798, Singapore; lisz@ntu.edu.sg

**Keywords:** viscoelastic fluid, elastic instability, microfluid, direction-dependent

## Abstract

This paper reports flow direction-dependent elastic instability in a symmetry-breaking microchannel. The microchannel consisted of a square chamber and a nozzle structure. A viscoelastic polyacrylamide solution was used for the instability demonstration. The instability was realized as the viscoelastic flow became asymmetric and unsteady in the microchannel when the flow exceeded a critical Weissenberg number. The critical Weissenberg number was found to be different for the forward-directed flow and the backward-directed flow in the microchannel.

## 1. Introduction

In Newtonian fluid, flow complexity originates mostly from the nontrivial inertial effect, which is mainly induced in the macroscale condition [1]. In microfluidics, the Reynolds (*Re*) number of Newtonian fluid is usually a very small value, and the inertial effect is negligible. Therefore, only creeping flow is induced in the microscale Newtonian fluid [2]. For viscoelastic fluid, on the other hand, complex flow behavior can be stimulated from the polymer molecules or surfactant dispersed in the fluid [3,4,5]. In certain designed microchannel structures, the polymer molecules or surfactant can be compressed or stretched to induce significant normal stress in the fluid. As a result, various complex flows such as turbulent flow, Couette flow, or swirling flow can be obtained in viscoelastic fluid even at the micrometer scale [6,7,8,9]. Analytically, complex flow can be understood from the Navier–Stokes equation, whereby the nonlinearity of Newtonian fluid depends on the advective term in the equation, while viscoelastic fluid nonlinearity is also contributed by the rheological effects from the normal stress in the fluid [10], which can be applied for mixing [11,12] or sorting [13] in microfluidics.

In particular, the elastic instability of viscoelastic fluid in specially designed microfluidic channels has been intensively studied [14,15]. One classical geometry of the microfluidic channel is a cross-slot, which consists of two perpendicular intersecting straight channels [16,17,18]. In the cross-slot, a stagnation point can be found at the cross center when fluid is injected into both ends of one straight channel and flows out from both ends of the other straight channel. Due to the structure symmetry, the flow velocity is 0 at the stagnation point while the velocity gradient is finite. This introduces an extensional flow in the microchannel, which can stretch or compress the polymer molecule in the fluid. The normal stress is, thus, induced and leads to elastic instability of the flow when the Weissenberg (*Wi*) number of the fluid exceeds a critical value. Below the critical *Wi* number, the viscoelastic flow is steady and symmetric, behaving as a Newtonian fluid. The flow pattern becomes asymmetric as the flow rate and *Wi* number increase beyond a critical vale. As the *Wi* number further increases, the flow pattern fluctuates and becomes time-dependent. Numerically, elastic instability was studied by investigating the flow of an upper-convicted Maxwell (UCM) fluid in a cross-slot channel, and different rheological conditions were analyzed for different instability types [19]. The Oldroyd-B model and a simplified Phan–Thien–Tanner model were also established for the analysis [20,21,22]. In addition, elastic instability has been demonstrated experimentally using different types of viscoelastic fluids such as polymer solution or micellar solution [23,24]. Instability was also studied by analyzing the flow under different aspect ratios of the cross-slot geometry [19]. The cross-slot was also used as a flow-focusing device to induce purely elastic instability [25]. Other geometries, such as a T-structured channel, were proposed, and a direct transition from symmetric flow to time-dependent flow was observed in the channel [26]. Contraction–expansion microchannels were also proposed, in which the extension and relaxation of polymer molecules were observed to study the elastic instability [27].

Previous work has mainly focused on the effect of microstructure geometry and fluid properties on the flow elastic instability. In these studies, the instability was fully characterized by the Weissenberg number. Here, we experimentally demonstrate a flow direction-sensitive elastic instability even at the same Weissenberg number. This can be used to stabilize or induce elastic instability by simply altering the flow direction without changing the flow rate. Unlike previous designs, the microchannel was a symmetry-breaking geometry, consisting of a square chamber and a nozzle structure. Therefore, the flow path in one direction was not the same as that in the opposite direction. This microchannel with carefully designed asymmetry has been intensively reported to realize a rectifying property, which induces different flow pressure depending on the flow direction under the same flow rate [28,29]. The microfluidic diode and memory were then developed on the basis of this rectifying function [30,31]. Here, we observed flow direction-dependent elastic instability, with symmetric steady flow evolving into an asymmetric unsteady flow. In addition, turbulence was also observed in certain *Wi* number conditions in the unsteady flow.

## 2. Methods

**Figure 1:**
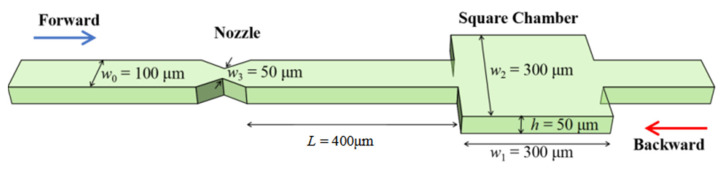
Schematic of microchannel consisting of a square chamber and nozzle structure.

The microchannel applied for obtaining non-Newtonian fluid instability was fabricated using a standard soft lithography process. A SU8-3000 photoresist (MicroChem) layer with a thickness of 50 µm was first spin-coated on a Si wafer, followed by an optical lithography process to develop the microchannel pattern. Liquid PDMS with a base and curing agent mixing ratio of 10:1 was then spin-coated on the patterned SU8 layer and heated for 4 h at 65 °C, yielding a solid layer of 50 µm thickness. The solid PDMS layer was peeled off from the Si wafer and bonded with a flat PDMS layer. The fabricated microfluidic channel is illustrated in Figure 1. The channel was composed of a square chamber and a symmetric nozzle structure. The square chamber in the microfluidic channel induces expansion and contraction of the flow, which introduces normal stress into the viscoelastic fluid. The nozzle structure is located on one side of the square chamber, which increases the localized flow resistance and confers the whole microfluidic channel with a symmetric-breaking structure. As shown in Figure 1, the width and height of the microchannel were *w*_0_ = 100 μm and *h =* 50 μm, respectively. The width and length of the square chamber were *w*_1_ = 300 μm and *w*_2_ = 300 μm, and the nozzle had a width of 50 μm. The channel between the left-side inlet and the nozzle structure, and the channel between the right-side inlet and the square chamber were both designed with a length of 4 mm, which was long enough for the full development of the flow. To clarify the two flow directions in the microchannel, the forward direction was defined as the direction with flow passing through the nozzle first, while the backward direction was defined as the direction with flow passing through the square chamber first, as indicated by the blue arrow and red arrow in Figure 1, respectively.

The streamline of the flow was recorded under a microscope by adding polystyrene tracking particles with a diameter of 1 µm in a concentration of 0.6 µL/mL. The flow pattern was studied at different flow rates and, therefore, different *Wi* numbers for the viscoelastic fluid. The Weissenberg number, defined as Wi=λU/L, describes the elasticity of the flow and qualifies the nonlinearity of the fluid, where L is the characteristic length of the channel, U is the flow rate in the channel, and λ is the relaxation time of the fluid, referring to the characteristic stretch–relax time of the polymer, which was measured as ~0.1 s for the PAM of 500 ppm. The Reynolds number is used to characterize the relationship between the inertial and viscous forces in the Newtonian fluid, expressed as Re=ρUL/η, where ρ is the density of the fluid.

## 3. Results and Discussion

**Figure 2:**
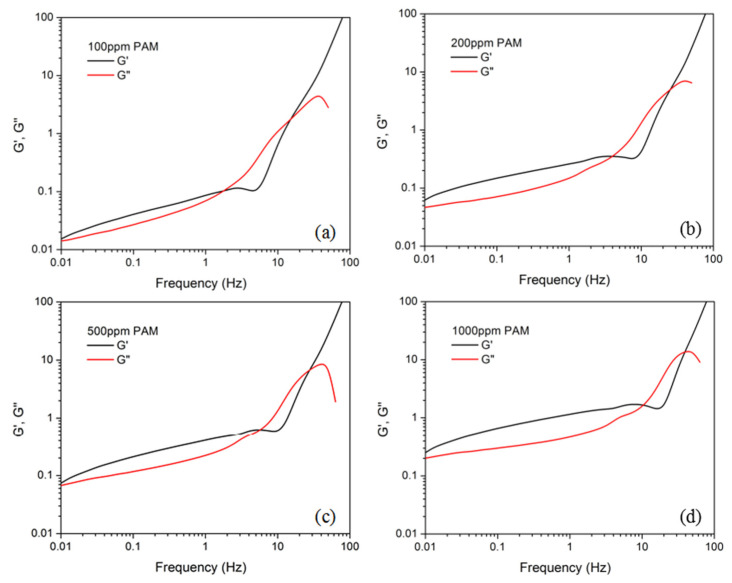
Complex shear modulus of the PAM solutions of (**a**) 100, (**b**) 200, (**c**) 500, and (**d**) 1000 ppm.

**Figure 3:**
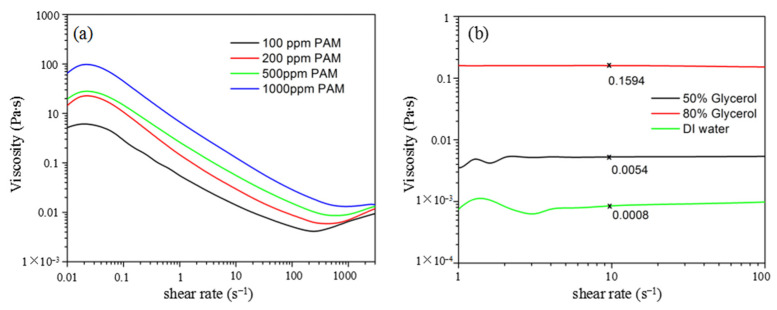
(**a**) Viscosity of non-Newtonian PAM solution and (**b**) Newtonian solutions.

We used polyacrylamide (PAM) with a molecular weight of 18 million for the viscoelastic fluid instability demonstration. PAM of 100, 200, 500, and 1000 ppm was measured using a rotational rheometer (Malvern, Discovery HR1) with cone-plate geometry. The cone had a diameter of 60 mm and angle of 2.006°. The complex shear modulus of the PAM solutions are shown in Figure 2. The storage modulus G′ was larger than the loss modulus G″ at the lower frequency band, confirming the elastic property of the fluid. The two curves of G′ and G″ crossed at 2, 4, 5, and 10 Hz for PAM of 100, 200, 500, and 1000 ppm. An increased crossing frequency indicates a larger elasticity of the solution with a higher ppm value. The viscosity of the fluid at different shear rates is shown in Figure 3a. The viscosity continuously decreased from ~10 Pa∙s to ~0.01 Pa∙s in the shear rate range from 0.1 s^−1^ to 100 s^−1^. For comparison, the viscosity of two typical Newtonian fluids, glycerol solution and DI water, was also measured, as shown in Figure 3b. The viscosity remained almost constant at 0.1595 and 0.0054 Pa∙s for the 80% and 50% volume concentrations of glycerol, respectively, and at 0.0008 Pa∙s for the DI water. For the subsequent fluid instability investigation, we chose PAM of 500 ppm with moderate viscosity.

**Figure 4:**
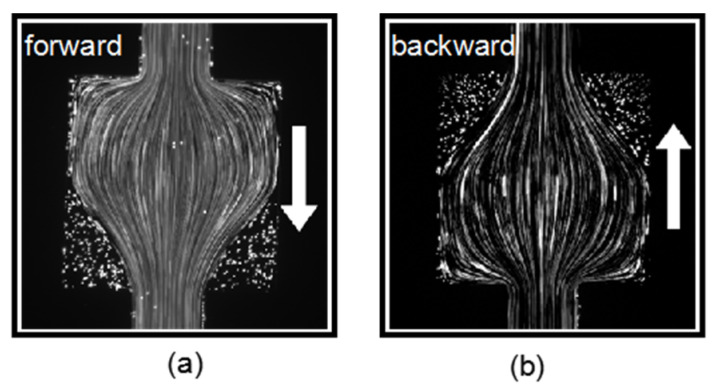
Viscoelastic flow pattern in the square chamber for flow along (**a**) the forward direction and (**b**) the backward direction at *Wi* = 5.56.

The flow of the PAM solution in the nozzle–square microchannel was first investigated in low *Wi* number conditions. The flow rate was controlled with a syringe pump (NE-300 Just Infusion™). It was first set to *Q =* 100 µL/h, corresponding to *Wi* = 5.56. Figure 4a,b present the streamline patterns of the forward flow and backward flow in the square chamber, respectively. The streamline patterns were both symmetric and steady in the low Weissenberg condition for the forward and backward flow, indicating that no elastic instability was induced. The flow patterns for both directions were almost the same; therefore, the nozzle structure has little impact on the flow in low *Wi* number conditions. For both directions, the streamline expanded to the square chamber first when entering the contraction–expansion structure, and then gradually concentrated to the center with a small streamline curvature. On the other hand, at the two corners of the chamber where the fluid flowed out, the tracking particles remained static and the fluid formed a stationary regime.

**Figure 5:**
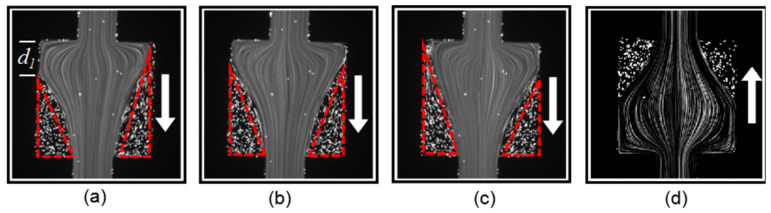
Viscoelastic flow pattern in the square chamber for flows along the forward direction at (**a**–**c**) three different instants, and (**d**) backward direction at *Wi* = 16.67.

Then, we increased the flow rate of PAM solution to *Q =* 300 µL/h, and the *Wi* number was increased to 16.67. The patterns recorded at three different instants are shown in Figure 5a–c. Similarly, to Figure 4a, the streamline expanded to the square chamber first when entering the contraction–expansion structure, and then gradually concentrated to the center. However, the curvatures of the streamline on the left side and the right side of the flow were no longer always the same. In addition, the stationary triangle regime (highlighted by the red dashed line) increased in size compared to Figure 4a. It can be clearly seen that the forward flow pattern became asymmetric and time-dependent. As the inertial effect is negligible at this flow rate, the instability should be purely elastic, thus stemming from the normal stress in the viscoelastic fluid. On the other hand, the backward flow at the same flow rate and *Wi* number, as shown in Figure 5d, remained steady and symmetric, and the flow pattern was almost the same as that in Figure 4b. The significant difference between the forward and backward flow patterns indicates that the flow elastic instability was direction-dependent in the asymmetric microchannel structure. For forward flow, it became extensional at the nozzle structure, and the normal stress increased before the fluid entered the square chamber. As a result, it was more likely to induce elastic instability. On the other hand, for backward flow, the nozzle structure increased the flow resistance of the fluid passing through the square chamber, which stabilized the flow.

**Figure 6:**
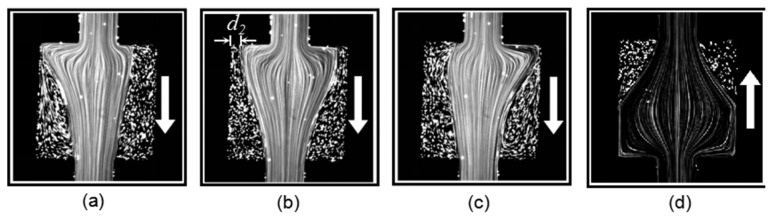
Viscoelastic flow pattern in the square chamber for flows along the forward direction at (**a**–**c**) three different instants, and (**d**) backward direction at *Wi* = 55.56.

As the flow rate of the PAM solution was increased to 1000 µL/h, the *Wi* number increased to 55.56. The instability of the forward flow became more significant, as shown in Figure 6a–c, which represent the flow pattern at different instants. In Figure 6a, the streamline was biased to the left side, and a large stationary regime formed on the right side of the square chamber. In particular, due to the large normal stress, turbulence occurred in the left-side triangle regime. In Figure 6b, the flow became symmetric and the turbulence disappeared. When the streamline become right-side-biased, as shown in Figure 6c, turbulence formed again in the right-side triangle regime. The streamlines in Figure 6a,c are almost mirror images. On the other hand, for backward flow, as shown in Figure 6d, the flow was still stabilized by the nozzle structure and remained symmetric and steady.

**Figure 7:**
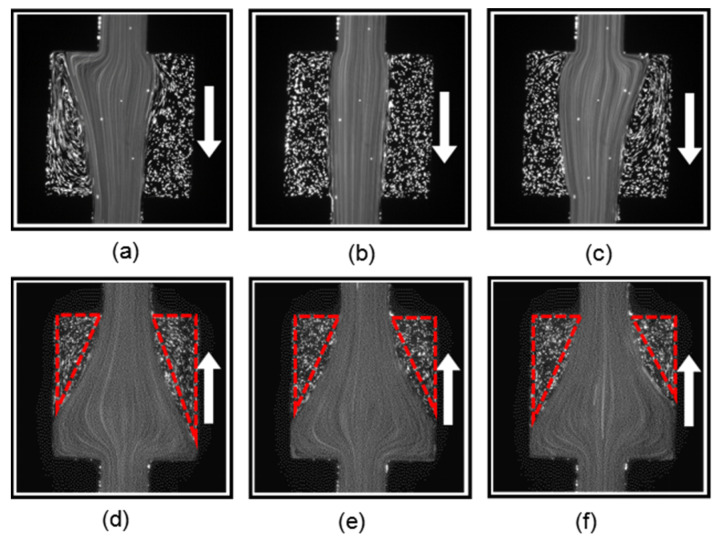
Viscoelastic flow pattern in the square chamber for (**a**–**c**) flows along the forward direction at three different instants, and (**d**–**f**) flows along the backward direction at three different instants at *Wi* = 111.11.

As the flow rate of the PAM solution was increased to 2000 µL/h, the *Wi* number increased to 111.11. The forward flow was still asymmetric and unsteady with turbulence on the streamline biased side, as shown in Figure 7a–c. On the other hand, as shown in Figure 7d–f, the backward flow started to become asymmetric and unsteady in this high *Wi* number condition. The increase in elastic instability in the backward flow was due to the normal stress now being large enough to exceed the stabilizing effect by the nozzle structure.

**Figure 8:**
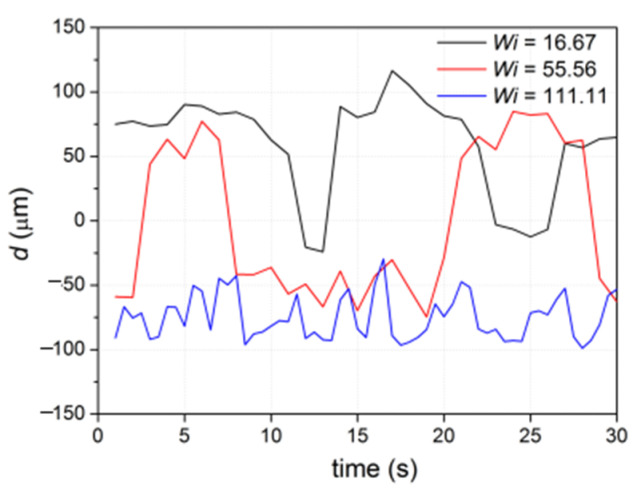
The edge length of the forward-flowing streamline on the left side of the flow.

To quantitatively analyze the instability behavior, we measured the length between the left upper corner of the square chamber and the left edge of the forward-flowing streamline. We noted that length *d* = *d*_1_ (as in Figure 5a) when the streamline edge was on the left side of the chamber, and *d* = −*d*_2_ (as in Figure 6b) when the streamline edge was on the upper side of the chamber. The real-time change of *d* from 1 s to 30 s is plotted in Figure 8 for different *Wi* numbers. We can see obvious resonances of *d* for the cases *Wi* = 16.67 and 55.56, indicating a significant instability of the streamline. The streamline continuously oscillated with a period of ~10 s and ~20 s for *Wi* = 16.67 and 55.56, respectively. In addition, *d* was mostly above 0 for *Wi* = 16.67, indicating that the edge of the streamline was on the left-side edge of the square chamber. On the other hand, *d* fluctuated between positive and negative values for *Wi* = 55.56, indicating that the edge of the streamline was on the left-side edge and upper-side edge of the square chamber, respectively. When *Wi* = 111.11, *d* was a negative value because the edge of the streamline remained on the upper-side edge of the square chamber at all measured timepoints. Thus, the oscillation became less obvious.

**Figure 9:**
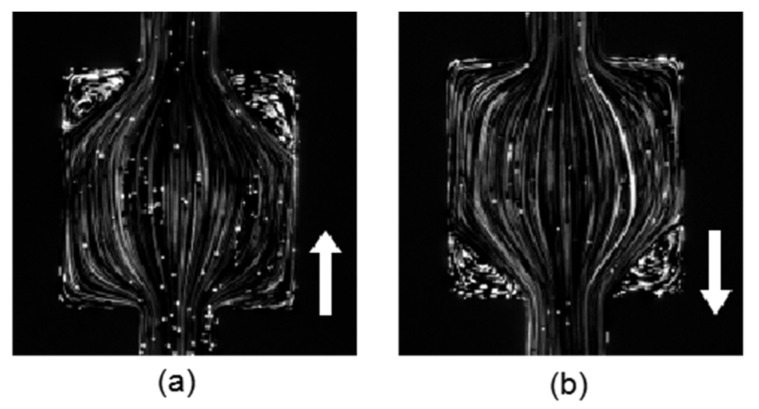
Flow pattern of the glycerol solution in (**a**) forward direction and (**b**) backward direction at *Q =* 2000 µL/h.

To verify whether the above instability was elastic-related, we injected Newtonian fluid into the nozzle–square microchannel for comparison. Here, a glycerol solution of 80% volume concentration is used. The flow rate was set to *Q =* 2000 µL/h, corresponding to an *Re* number of 0.07. As shown in Figure 9a,b, the forward flow and backward flow of the glycerol solution were both symmetric and steady, and both flow patterns were almost the same. This verifies that the asymmetric flow pattern, the unsteady streamline, and the flow direction-dependent instability of the PAM solution were due to the fluid elastic property.

**Figure 10:**
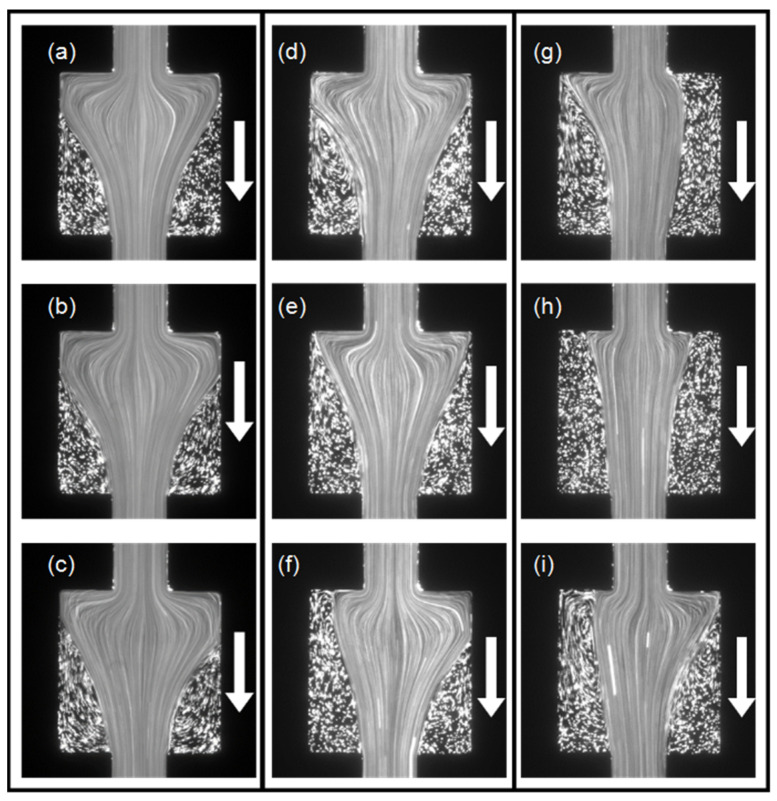
The PAM flow pattern in the symmetric square-only microchannel at three different instants (**a**–**c**) when *Wi* = 16.67, (**d**–**f**) when *Wi* = 55.56, and (**g**–**i**) when *Wi* = 111.11.

To further explain the effect of the nozzle structure, we prepared a microchannel without a nozzle structure using only the same square chamber. The microchannel structure was symmetric; thus, we only investigated the PAM flow along one direction. Figure 10a–c show the flow pattern of the PAM solution at different instants at *Q =* 300 µL/h and *Wi* = 16.67. The flow remained in a symmetric static state, and there was no flow instability. Figure 10d–f show the flow pattern of the PAM solution at different instants at *Q =* 1000 µL/h and *Wi* = 55.56. The flow became asymmetric and unsteady due to the increased normal stress when the flow entered the expansion square chamber and formed an extensional flow. As the flow rate *Q* increased to 2000 µL/h and *Wi* = 111.11, the asymmetry and instability of the flow pattern became more obvious, as presented by the three different patterns shown in Figure 10g–i.

**Figure 11:**
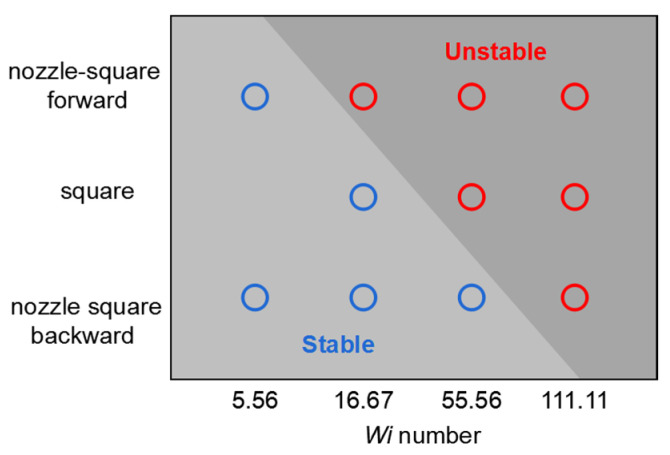
Critical Weissenberg number phase diagram for the two flow directions in the nozzle–square microchannel and the flow in the square-only microchannel.

On the basis of the above analysis, the instability of the viscoelastic flow in the asymmetric nozzle–square microchannel and in the symmetric square microchannel is summarized in Figure 11. The critical *Wi* number for the flow instability was not only different for the square–nozzle microchannel and the square-only microchannel, but also different for the forward-directed flow and the backward-directed flow in the same nozzle–square microchannel. The critical *Wi* number for the forward-directed flow in the nozzle–square microchannel was the smallest, indicating that it is easiest to induce elastic instability in such a flow condition. This is because the polymers in the viscoelastic fluid were stretched by the nozzle structure before entering the square structure, facilitating the induction of normal stress in the subsequent extensional flow. On the other hand, the critical *Wi* number for the backward-directed flow in the nozzle–square microchannel was the largest, indicating that it is hardest to induce elastic instability in such a flow condition. This is because the nozzle structure increased the flow resistance before the flow entered the square structure, which stabilized the flow.

## 4. Conclusions

In summary, we studied the viscoelastic fluid instability in an asymmetric nozzle–square microchannel. The instability was demonstrated to be purely elastic and dependent on the flow Weissenberg number. Beyond a certain *Wi* number, the flow pattern was converted from a symmetric steady state to an asymmetric unsteady state. The critical *Wi* number was demonstrated to be different for the two flow directions in the same nozzle–square microchannel. In other words, the flow instability was flow direction-dependent even at the same *Wi* number and in the same microchannel structure. This flow direction-dependent instability can not only be applied for fluidic rectifier applications, but also be used to stabilize viscoelastic flow in high-flow-rate conditions for mass transportation.

## Data Availability

Raw data presented in this study are available on request from the corresponding author.
